# Supplementary material for: Post-discharge mortality in suspected pediatric sepsis: Insights from rural and urban healthcare settings in Rwanda
Source: PLOS Glob Public Health. 2025 Dec 11;5(12):e0005622. doi: 10.1371/journal.pgph.0005622 (PMC12697939; doi:10.1371/journal.pgph.0005622)
Supplement: S1 Table — Overall demographics and cohort characteristics. (DOCX) [file pgph.0005622.s002.docx]

**S1 Table: Supplementary Table 1**

|  | **N = 1127** | |
| --- | --- | --- |
| **Variables** | **N (%)/Median (IQR)** | **aOR* (95% CI)** |
| **Site, n (%)** |  |  |
| Kigali | 361 (32.3) | reference |
| Ruhengeri | 766 (68.0) | 0.24 (0.14-0.43) |
| **Male (ref=female)** | 676 (60.0) | 0.81 (0.47-1.39) |
| **Age, months** | 13.5 (6.1-24.7) | 0.96 (0.94-0.99) |
| **MUAC (mm)^1^** |  |  |
| <110/<115 | 109 (9.7) | 2.69 (1.19-6.08) |
| 110-120/115-125 | 112 (9.9) | 3.01 (1.47-6.17) |
| >120/ >125 | 906 (80.4) | reference |
| **Weight for age z-score** |  |  |
| <-3 | 103 (9.1) | 4.63 (2.42-8.85) |
| -3 to -2 | 116 (10.3) | 2.62 (1.22-5.65) |
| >-2 | 908 (80.6) | reference |
| **SpO_2_, %** | 95 (88-97) | 0.98 (0.95-1.02) |
| **Heart rate** | 142 (128-157) | 1.00 (0.99-1.02) |
| **Respiratory rate** | 41 (35-49) | 1.01 (0.99-1.03) |
| **Temperature** | | |
| < 36.5 | 208 (18.5) | 0.75 (0.34-1.66) |
| 36.5-37.5 | 419 (37.2) | reference |
| >37.5 | 500 (44.4) | 0.67 (0.37-1.22) |
| **Abnormal BCS** | 194 (17.2) | 1.93 (1.06-3.52) |
| **HIV positive** | 3 (0.3) | 4.04 (0.35-46.89) |
| **Positive malaria test** | 17 (1.5) | - |
| **Hemoglobin, g/dl** | | |
| No anemia: ≥ 11 | 712 (63.2) | reference |
| Anemic: < 11 | 415 (36.8) | 1.74 (1.00-3.00) |
| **Respiratory distress** | 228 (20.2) | 1.37 (0.76-2.48) |
| **Referral** | 1010 (89.6) | 2.37 (0.90-6.24) |
| **Prior antibiotic use** | 423 (37.5) | 1.58 (0.80-3.11) |
| **Prior antimalarial use** | 33 (2.9) | 1.98 (0.63-6.23) |
| **Maternal and Social Characteristics** | | |
| **Time to reach hospital** | | |
| <30 min | 453 (40.2) | reference |
| 30 min - 1h | 438 (38.9) | 1.28 (0.63-2.60) |
| >1h | 236 (20.9) | 1.88 (0.91-3.88) |
| **Maternal education^2^** | | |
| No school or <=P3 | 151 (13.4) | reference |
| P4 to P6 | 457 (40.6) | 0.46 (0.23-0.91) |
| S1 to S6 | 428 (38.0) | 0.24 (0.11-0.52) |
| > S6 | 88 (7.8) | 0.13 (0.04-0.49) |
| **Water source** |  |  |
| Municipal water/tap | 715 (63.4) | reference |
| Other sources | 412 (36.6) | 1.33 (0.76-2.30) |
| **Boil/disinfect/filter water** | 441 (39.1) | 0.46 (0.24-0.88) |
| **Discharge Characteristics** | | |
| **Discharge status** | | |
| Routine discharge | 1097 (97.3) | reference |
| Referred to higher level of care | 21 (1.9) | 2.77 (0.85-9.01) |
| Unplanned discharge | 9 (0.8) | 1.57 (0.18-13.50) |
| **Length of stay** | 4 (3-8) | 1.06 (1.04-1.09) |

Note: *adjusted for age, sex, and site. ^1^small number represents cutoff for under 6 months; ^2^3 participants reported unknown level of eduation

Abbreviations: OR = odds ratio; IQR = interquartile range; BCS = Blantyre Coma scale; HIV = human immunodeficiency virus; SpO_2_ = oxygen saturation
