# Supplementary material for: Post-discharge mortality in suspected pediatric sepsis: Insights from rural and urban healthcare settings in Rwanda
Source: PLOS Glob Public Health. 2025 Dec 11;5(12):e0005622. doi: 10.1371/journal.pgph.0005622 (PMC12697939; doi:10.1371/journal.pgph.0005622)
Supplement: Attachment — Submitted filename: Reviewers comments and authors response_CU_v2.docx [file pgph.0005622.s004.docx]

**Reviewers’ comments and Authors’ responses**

**Comment 1:**
*“The journal format and technical production system, as well as the ethics of scientific research, must be adhered to. In addition, I want to know if the mortality rate was due to malnutrition and was this the main reason?”*

**Response:**
We appreciate this observation. We’ve reviewed the manuscript to ensure it aligns fully with the journal’s technical and ethical standards. Regarding malnutrition, we’ve clarified its role as a significant—but not sole—predictor of post-discharge mortality. This is now explicitly addressed in the Results (Lines 246–247) and expanded on in the Discussion (Lines 272–288).

**Comment 2:**
*“Formatting of Data Access Statement: Ensure the font size, style, and alignment of the Data Access Statement match the required format of the journal. The placement of this statement should follow the journal’s guidelines—typically under a separate section titled "Data Availability" or "Data Access."Since the citation style (e.g., "[1]" and "[2]") is correct, no changes are needed there. However, ensure that references are formatted consistently in the reference list”*

**Response:**
Thank you for pointing this out. The Data Availability section has been reformatted and moved to its appropriate position in the manuscript (Lines 353–362). We also reviewed the reference list for consistency in formatting. References previously cited as [1] and [2] are now [16] and [38] due to updated ordering.

**Comment 3:**
*“The conclusion does not fully align with the study’s objectives and findings. It should directly reflect the study’s key results and not introduce new ideas that were not analyzed. The authors should explicitly link their findings to the research objectives to maintain coherence. The conclusion should not only summarize findings—it must include specific, actionable recommendations based on the study’s results. Examples of improvements:*

- *Strengthening post-discharge monitoring programs for at-risk pediatric patients.*
- *Implementing targeted nutritional support interventions for children with malnutrition.*
- *Developing policies to improve healthcare access in socially vulnerable populations.*

*The recommendations should clearly state who (healthcare providers, policymakers, NGOs, etc.) should implement them and how they can be applied in practice.”*

**Response:**
We agree. The Conclusion has been rewritten to closely reflect our findings and objectives. We've added actionable recommendations directed at relevant stakeholders—especially healthcare providers and policymakers—including strengthening follow-up systems, improving in-hospital nutritional support, and addressing structural barriers to care (Lines 337–351).

**Comment 4:**
*“Justification of the Study: The rationale for conducting the study should be more explicitly stated in the introduction and discussion sections. The authors should clearly explain:*

- *Why this research was necessary (e.g., gaps in knowledge, limited data in Rwanda, policy implications).*
- *How their findings contribute to existing literature and why this study is significant.*
- *What impact the study has on clinical practice, policy, or future research directions.”*

**Response:**
Thank you. We revised the Introduction to better highlight the knowledge gaps and the need for data on post-discharge outcomes in Rwanda (Lines 96–108). In the Discussion (Lines 310–315), we now clearly link our findings to existing literature and emphasize their relevance for clinical care, policy, and future research in similar low-resource settings.

Thank you again for your helpful comments. These lines are cited based on the Manuscript without track changes. We believe these revisions have strengthened the manuscript, and we hope it will now meet the journal’s expectations.

Sincerely,

**Dr. Christian Umuhoza**
(On behalf of all co-authors)
Consultant Pediatrician, University Teaching Hospital of Kigali
Senior Lecturer, University of Rwanda
