# Supplementary material for: Post-discharge mortality in suspected pediatric sepsis: Insights from rural and urban healthcare settings in Rwanda
Source: PLOS Glob Public Health. 2025 Dec 11;5(12):e0005622. doi: 10.1371/journal.pgph.0005622 (PMC12697939; doi:10.1371/journal.pgph.0005622)
Supplement: Attachment — Submitted filename: Response to Reviewers (2).docx [file pgph.0005622.s006.docx]

# Response to Reviewers

Dear Editor and Reviewers,

We thank you for your thorough review of our manuscript titled “Post-discharge mortality in suspected pediatric sepsis: insights from rural and urban healthcare settings in Rwanda” (Manuscript ID: PGPH-D-24-02667R1). We greatly appreciate your constructive feedback, which has helped us to significantly improve our manuscript. Please find below our point-by-point responses to each of the comments raised. We have indicated the corresponding changes and provided relevant line numbers in the revised manuscript.

## Abstract

**Reviewer comment:**

The abstract lacks keywords and it lacks objective as well. The main objective is missing in the abstract.

**Response:**

Thank you for this helpful suggestion. We have now added a clear objective to the abstract, along with a set of keywords, to improve clarity and completeness. Please see lines 69–70 for the changes. (We added the following information: Keywords: Post-discharge mortality; Pediatric sepsis; Low- and middle-income country; Risk prediction; Health system access.)

## Introduction

**Reviewer comment:**

Line 64 and 65 lack intext citation. Authors should kindly re-write their introduction again to suit their topic. There is no gap found in the introduction. Authors should let their reader know the gaps they identified.

**Response:**

We appreciate this feedback. The introduction has been revised to better align with the study focus. Additionally, in-text citations have been included where needed. We now clearly articulate the knowledge gap in lines 99–111, emphasizing the lack of Rwandan data on post-discharge mortality and the need for locally relevant evidence.

## Methods

**Reviewer comment:**

The methods lacks details element including study population, sample size determinants, inclusion and exclusion and sample method.

**Response:**

Thank you for this observation. We now clarify that the study was prospective, enrolling all eligible children within the age group and criteria described in lines 118–127. Inclusion and exclusion criteria are detailed in lines 133–137. We also note that our goal was to recruit a minimum of 1,000 children to ensure sufficient power for our analysis, as described in lines 176–178.

## Results

**Reviewer comment:**

Table 1: Cohort characteristics and disposition, stratified by ages 0-6 months and 211 ages 6-60 months. What are the authors reporting, regression ? descriptive ? please check the interpretation.

**Response:**

Thank you for raising this point. Table 1 includes both descriptive statistics (frequencies, percentages, and medians with IQRs) and adjusted odds ratios (aORs) obtained from multivariable logistic regression models assessing associations with post-discharge mortality. To make this clearer, we have updated the table title (lines 234–235) and added a caption note to indicate that the aORs were adjusted for age, sex, and study site.

We hope that our revisions have fully addressed your concerns. Thank you once again for your valuable time and constructive input toward enhancing the quality of our work.

Sincerely,

**Christian Umuhoza,** MD, MMED
On behalf of all co-authors

Centre Hospitalier Universitaire de Kigali
[crissumuh@gmail.com](mailto:crissumuh@gmail.com), [christian.umuhoza@chuk.rw](mailto:christian.umuhoza@chuk.rw)
