# Supplementary material for: Post-discharge mortality in suspected pediatric sepsis: Insights from rural and urban healthcare settings in Rwanda
Source: PLOS Glob Public Health. 2025 Dec 11;5(12):e0005622. doi: 10.1371/journal.pgph.0005622 (PMC12697939; doi:10.1371/journal.pgph.0005622)
Supplement: Attachment — Submitted filename: PGPH-D-24-02667R2.docx [file pgph.0005622.s005.docx]

**Post-discharge mortality in suspected pediatric sepsis: insights from rural and urban healthcare settings in Rwanda**

Thank you for the opportunity to review this important and timely research that addresses a critical and under-researched topic.The topic is highly relevant to sub-Saharan Africa, and the lack of prior studies in Rwanda makes this contribution both novel and valuable for informing national healthcare priorities. I Thank the authors for their efforts in conducting a prospective, multisite study and for highlighting the rural-urban disparities in outcomes in Rwanda.

**General:**

Please improve some grammar and a concise in objective narrative of the manuscript.

**Abstract:**

1. Title and Abstract Consistency:
    The title refers specifically to "suspected pediatric sepsis", but the abstract does not clearly define how sepsis was identified or suspected. Clarifying this would help contextualize the findings.
2. While it is understood that post-discharge causes of death can be difficult to verify, the abstract would benefit from the inclusion of any available information on probable or leading cause-specific mortality, even if based on caregiver reports or verbal autopsy methods.
3. Data clarity: Please clarify the group age range being reported on. For example
    "Children aged <2 months exhibited the highest cumulative mortality risk."
    This is confusing, as the age cohort is 0–6 months. Does this refer to a sub-analysis within the 0–6-month cohort? Clarify or remove if not directly supported by data.
4. Improvement on conclusion: The conclusion emphasizes the need for targeted post-discharge interventions, but it would be stronger if it briefly mentioned what kinds of interventions might be most feasible or effective in the Rwandan context mostly what ou saw was successful while tring to prevet death.

Manuscript:

1. Method section
   a. Please improve on how ou defined “suspected infection” was operationalized.
   b. Please specift why specific age bands were chosen for Kaplan-Meier analysis.
   c. What was followed during verbal autopsy? This should be briefly described or cited. Was it validated in rwandan context? Is it from WHO?
2. Results section
   a. Consider using a consolidated flow diagram or summary table (e.g., CONSORT-style) for clarity.
   b. Clarify whether readmissions were to the same facility or any healthcare facility, and whether diagnoses at readmission were tracked.
3. Discussion
   a. The findings are summarized well; however, the discussion section could benefit from more literature explaining why certain factors presented in the results may contribute to post-discharge mortality, including the noted gap in follow-up visits. Is this gap unique to Rwanda, or is it also observed in other LMICs?
   b. This research could inform policymakers and pediatricians in establishing effective discharge planning. Please Consider adding a call to action for health system strengthening in similar settings within Rwanda and across sub-Saharan Africa.
