# Supplementary material for: Post-discharge mortality in suspected pediatric sepsis: Insights from rural and urban healthcare settings in Rwanda
Source: PLOS Glob Public Health. 2025 Dec 11;5(12):e0005622. doi: 10.1371/journal.pgph.0005622 (PMC12697939; doi:10.1371/journal.pgph.0005622)
Supplement: Attachment — Submitted filename: Response to Reviewers Letter V2.docx [file pgph.0005622.s007.docx]

# Response to reviewers letter

| **Reviewer Comment** | **Author Response** | **Line Numbers in Revised Manuscript** |
| --- | --- | --- |
| Please improve some grammar and a concise in objective narrative of the manuscript. | We revised the manuscript throughout for grammar, clarity, and conciseness. The Abstract, Introduction, Methods, and Discussion were carefully streamlined. |  |
| The title refers specifically to "suspected pediatric sepsis", but the abstract does not clearly define how sepsis was identified or suspected. Clarifying this would help contextualize the findings. | We clarified that children were included if admitted with suspected or confirmed infection, as assessed by the treating clinician. | 48-49 |
| While it is understood that post-discharge causes of death can be difficult to verify, the abstract would benefit from the inclusion of any available information on probable or leading cause-specific mortality, even if based on caregiver reports or verbal autopsy methods. | We sincerely apologize for the earlier inaccurate statement. Verbal autopsies were not performed in this study. The error arose because our group has used the WHO 2022 Verbal Autopsy (VA) tool in Uganda, and that language inadvertently carried over. We corrected this and clarified that although the tool exists in Rwanda, VA was outside the scope of this study and remains an area for future research. |  |
| Data clarity: Please clarify the group age range being reported on. For example "Children aged <2 months exhibited the highest cumulative mortality risk." This is confusing, as the age cohort is 0–6 months. Does this refer to a sub-analysis within the 0–6-month cohort? Clarify or remove if not directly supported by data. | We clarified that this refers to a sub-analysis within the 0–6 month cohort. | 62–63 |
| Improvement on conclusion: The conclusion emphasizes the need for targeted post-discharge interventions, but it would be stronger if it briefly mentioned what kinds of interventions might be most feasible or effective in the Rwandan context mostly what ou saw was successful while tring to prevet death. | We revised the Abstract conclusion to specify feasible interventions: “caregiver counselling, scheduled follow-up visits, and community health worker monitoring.” | 64-66 |
| Please improve on how you defined “suspected infection” was operationalized. | We clarified that suspected infection was defined by the treating clinician’s assessment of suspected or confirmed infection, consistent with prior Ugandan studies. | 132-137 |
| Please specify why specific age bands were chosen for Kaplan-Meier analysis. | We added justification: the age bands were chosen to reflect developmental and immunological stages, align with pediatric sepsis literature, and enable comparison with other studies. | 193-198 |
| What was followed during verbal autopsy? This should be briefly described or cited. Was it validated in rwandan context? Is it from WHO? | We corrected the oversight. Verbal autopsies were not conducted. The revised Methods state: “Although the WHO 2022 standard VA tool, adapted for Rwanda, was available, VA was outside the scope of this work.” | 174-179 |
| Consider using a consolidated flow diagram or summary table (e.g., CONSORT-style) for clarity. | Figure 1 faithfully summarize enrollment, follow-up, and outcomes. | Figure 1 reference at line 227 |
| Clarify whether readmissions were to the same facility or any healthcare facility, and whether diagnoses at readmission were tracked. | We clarified that readmissions included any healthcare facility (per caregiver report).  Diagnoses were not systematically tracked; this is now acknowledged as a limitation. | 278  355-356 |
| The findings are summarized well; however, the discussion section could benefit from more literature explaining why certain factors presented in the results may contribute to post-discharge mortality, including the noted gap in follow-up visits. Is this gap unique to Rwanda, or is it also observed in other LMICs? | We expanded the Discussion with examples from Uganda, Bangladesh, and systematic reviews showing that follow-up gaps are common across LMICs, not unique to Rwanda. | 308-314 |
| This research could inform policymakers and pediatricians in establishing effective discharge planning. Please Consider adding a call to action for health system strengthening in similar settings within Rwanda and across sub-Saharan Africa. | We added a call to action emphasizing integration of discharge planning into guidelines, strengthening CHW follow-up, use of HMIS, and multilateral partnerships. | 380-385 |
| Please ensure the conclusion underscores actionable steps. | The Conclusion now specifies: risk model development, scheduled follow-up for high-risk children, multilateral partnerships for transition-of-care, and broader health system strengthening. | 369-376 |
