# Supplementary material for: Post-discharge mortality in suspected pediatric sepsis: Insights from rural and urban healthcare settings in Rwanda
Source: PLOS Glob Public Health. 2025 Dec 11;5(12):e0005622. doi: 10.1371/journal.pgph.0005622 (PMC12697939; doi:10.1371/journal.pgph.0005622)
Supplement: Attachment — Submitted filename: Response to Reviewers Letter_13 Oct 2025 (1).docx [file pgph.0005622.s008.docx]

**Response to Reviewers**

Dear Editors and Reviewers,

We sincerely thank the reviewers and editors for their careful reading and constructive feedback on our manuscript. We greatly appreciate the time and expertise invested in improving the quality and clarity of our work. We have carefully considered each comment and made corresponding revisions throughout the manuscript. Below, we summarize our detailed responses and highlight how the requested changes were incorporated.

We are grateful that Reviewer #4 recognized the significant improvement in clarity, structure, and scientific rigor of the revised version, and we appreciate their recommendation for publication. We also thank Reviewer #5 for the insightful and detailed suggestions, which helped us clarify critical methodological aspects and strengthen the Discussion and Conclusions.

### **Response to Reviewer #5**

#### **1. Clarification on Verbal Autopsies (Lines 179-181)**

**Reviewer comment:** A contradiction exists between the previous response (that verbal autopsies were not performed) and the current statement indicating that they were performed to determine cause of death.

**Response:** We thank the reviewer for identifying this important inconsistency. We have now clarified that *verbal autopsies were not performed* as part of this study. While we adapted the WHO 2022 standard verbal autopsy tool for potential use, the conduct and analysis of verbal autopsies were **outside the scope** of this work.

**Revised text (Methods, “Variables and Measurements” section):**

“Although we did include the WHO 2022 standard verbal autopsy tool questions (adapted for Rwanda) for any participants who died following hospital discharge, in-depth verbal autopsy analysis was outside the scope of this study.”

This revision ensures methodological consistency and transparency regarding the data collected.

#### **2. Definition of ‘Suspected Sepsis’ (Lines 132-142)**

**Reviewer comment:** Please provide a more detailed operational definition or description of the clinical criteria guiding the treating clinician’s assessment of suspected sepsis.

**Response:** We fully agree that this clarification is essential. The definition has been expanded to explain that inclusion criteria were intentionally broad to reflect real-world diagnostic practices in low-resource settings, where clinicians primarily rely on clinical judgment.

**Revised text (Methods, “Participant recruitment and selection criteria” section):**

“Inclusion criteria included: any child within this age group admitted with suspected or confirmed infection, as assessed by the treating clinician. This was left to the discretion of the individual clinician and included any child being admitted with a diagnosis of at least one viral, bacterial, or parasitic infection based on clinical (e.g., tachypnea, hypoxia, fever with clinical diagnosis of pneumonia) or laboratory (e.g., rapid diagnostic test positive confirming diagnosis of malaria) criteria. Our previous research in similar settings in Uganda demonstrated that 90% of children enrolled using these same criteria for admission, with a confirmed or suspected infection based on the treating clinician’s assessment, met the International Pediatric Sepsis Consensus Conference criteria for sepsis.

#### **3. Definition of Abnormal Blantyre Coma Scale**

**Reviewer comment:** Please define what constitutes an abnormal Blantyre Coma Scale score.

**Response:** We have now clearly defined abnormal BCS as ≤4, consistent with previous pediatric sepsis studies.

**Revised text (Table 1 legend):**

“BCS ≤4 was considered abnormal.”

#### **4. Conclusions Section (Lines 368-399)**

**Reviewer comment:** Recommendations should be more tightly linked to statistically significant risk factors (malnutrition, abnormal BCS, travel time >1 hour, etc.).

**Response:** We agree and have revised the Conclusions to more explicitly connect our proposed actions with the key predictors identified in the results.

**Revised text (Conclusions):**

“These findings call for targeted interventions at discharge, including (i) routine nutritional screening and linkage to malnutrition support for all children with WAZ < -3, (ii) systematic early follow-up for children presenting with neurological impairment or abnormal Blantyre Coma Scale, and (iii) enhanced community follow-up through CHWs for children living more than one hour from health facilities. Such measures directly address the highest-risk groups identified in our analysis.”

This revision strengthens the translational relevance of the study’s conclusions.

#### **5. Discussion (Lines 290-352) – Urban vs. Rural Mortality**

**Reviewer comment:** Consider whether illness severity differed between sites or note if data were unavailable.

**Response:** We thank the reviewer for this thoughtful point. We have added a clarifying statement acknowledging the absence of illness severity data and recommending it as a focus for future investigation.

**Revised text (Discussion):**

“Baseline illness severity data (e.g., admission severity scores) were not available for comparison between sites. Future research incorporating standardized illness severity metrics at admission could clarify whether these differences reflect case-mix variations or systemic disparities.”

#### **6. Minor Edits and Formatting**

**Reviewer comment:** Proofread for grammatical accuracy and reformat tables (left-align variable names; center-align values).

**Response:** We performed a thorough grammatical and stylistic review using a professional grammar editor (as acknowledged in the cover letter). We also reformatted all tables according to journal style — variable names are now left-aligned and data values centered for improved readability.

### **Response to Reviewer #4**

**Reviewer comment summary:** Reviewer #4 commended the revisions, noting substantial improvement in clarity, quantitative rigor, and the strength of the Discussion.

**Response:** We are deeply grateful for Reviewer #4’s positive evaluation and encouraging remarks. We took their comments as affirmation that our revisions—particularly those that introduced actionable recommendations for healthcare providers, hospital leaders, and policy-makers—successfully enhanced the manuscript’s impact. No additional textual changes were required in response to this review.

### **Summary of Key Revisions**

| **Section** | **Key Change Implemented** |
| --- | --- |
| Methods | Clarified that verbal autopsies were *not performed*; expanded definition of suspected sepsis; defined abnormal Blantyre Coma Scale (≤4). |
| Discussion | Added note about lack of illness severity data between sites. |
| Conclusions | Linked actionable recommendations directly to identified risk factors (malnutrition, neurological impairment, travel time). |
| Tables | Adjusted alignment as requested. |
| Language & Readability | Comprehensive grammar and style revisions applied throughout. |

We believe these revisions have strengthened the manuscript substantially and improved its clarity, methodological transparency, and practical relevance. We once again thank the reviewers and editors for their valuable feedback and guidance.

Sincerely,

**Dr. Christian Umuhoza** (on behalf of all co-authors)

Department of Pediatrics,

University Teaching Hospital of Kigali

Email: [christian.umuhoza@chuk.rw](mailto:christian.umuhoza@chuk.rw)
